# On-farm analysis of exhaled breath compounds as indicators for a postpartum health score in dairy cattle: a case study

**DOI:** 10.1093/jas/skaf234

**Published:** 2025-07-18

**Authors:** Brigitte G C de Bruijn, Ingrid D E van Dixhoorn, Rudi M de Mol, Jeroen W F van Dongen, Jack C Musters, Joop T N van der Werf, István Fodor, Peter W G Groot Koerkamp, Cornelis G van Reenen

**Affiliations:** Wageningen Livestock Research, P.O. Box 338, 6700 AH Wageningen, The Netherlands; Wageningen Livestock Research, P.O. Box 338, 6700 AH Wageningen, The Netherlands; Wageningen Livestock Research, P.O. Box 338, 6700 AH Wageningen, The Netherlands; Relitech B.V., van Siburgstraat 34, 3864 HW Nijkerk, The Netherlands; Relitech B.V., van Siburgstraat 34, 3864 HW Nijkerk, The Netherlands; Wageningen Livestock Research, P.O. Box 338, 6700 AH Wageningen, The Netherlands; Wageningen Livestock Research, P.O. Box 338, 6700 AH Wageningen, The Netherlands; Wageningen University, Agricultural Biosystems Engineering, P.O. Box 16, 6700 AA Wageningen, The Netherlands; Wageningen Livestock Research, P.O. Box 338, 6700 AH Wageningen, The Netherlands

**Keywords:** dairy cow, exhaled breath composition, energy balance, postpartum disease, respiratory exchange ratio

## Abstract

The objective of this case study was to investigate if respiratory ratios derived from non-invasive exhaled breath measurements could be used as a tool to detect dairy cows at risk for impaired postpartum health. Measurements of exhaled breath from individual cows took place during visits to a concentrate feeder from 2 wk prepartum until 6 wk postpartum (Veterinary Metabolism System, Relitech). Per measurement, concentrations of oxygen (O_2_), carbon dioxide (CO_2_), and methane (CH_4_) (vol%) were measured. Subsequently, daily average respiratory exchange ratio (RER; CO_2_/O_2_), CH_4_ exchange ratio (MER; CH_4_/O_2_) and CH_4_-to-CO_2_ ratio (MCR; CH_4_/CO_2_) were calculated per cow. Cows were scored clinically twice weekly from 2 wk prepartum until 6 wk postpartum on 19 clinical signs. Blood β-hydroxybutyric acid was determined twice weekly, and additional blood samples were taken in week 1 and week 5 after calving to determine clinical-chemical parameters. A total deficit score (TDS) was assigned to each cow based on blood values and clinical scores. Per health score (e.g., decrease in body condition score (BCS) after calving, β-hydroxybutyric acid values, and TDS values), cows were divided into two categories (high or low). Differences in exhaled breath composition between these categories were evaluated using mixed models. The RER was lower in cows with a high reduction in BCS during the first 6 wk after calving, which can result in ketosis and fatty liver caused by the increased body fat mobilization. For TDS Locomotion, TDS Metabolic, TDS Liver, and TDS Macro-minerals, MER was lower in cows with a higher TDS compared with cows with a lower TDS, which may be related to decreased feed intake in cows at risk for disease leading to a decreased methane production. Prepartum MER was lower in cows with a high decrease in BCS, high TDS Total, or high TDS inflammation compared with cows with low values in these categories. The MCR was inconsistent for different disease categories. Based on the results of this experiment, respiratory ratios derived from non-invasive exhaled breath measurements seem to be promising indicators to detect cows at risk for disease around calving, but further validation of breath measurements is required. Variables such as individual daily milk production, dry matter intake, and time lag between breath measurement and feeding time should be taken into account in future research to improve the interpretation of results.

## Teaser

Non-invasive exhaled breath compounds are potential indicators to detect cows at risk for transition diseases.

## Introduction

In early lactation, 30% to 50% of dairy cows are prone to develop transition-related diseases, such as ketosis, fatty liver, digestive issues (e.g., displaced abomasum), macro-mineral disbalances (e.g., hypocalcemia), inflammatory diseases (e.g., endometritis, mastitis), or a combination of these (Sordillo and Mavangira, 2014; Sundrum, 2015; Wankhade et al., 2017). This increased susceptibility to diseases is linked to the metabolic and physiological challenges around parturition and the onset of lactation (LeBlanc, 2010; Wisnieski et al., 2019). In early lactation, many cows experience a negative energy balance (NEB), as feed intake is insufficient to meet the high energy requirements for milk production (Pushpakumara et al., 2003; Wathes et al., 2007; LeBlanc, 2010; Churakov et al., 2021). The postpartum NEB is marked by extensive lipid mobilization, which causes increased concentrations of non-esterified fatty acids (NEFA) and ketone bodies like β-hydroxybutyric acid (BHB) in the blood stream (Drackley, 1999). Elevated concentrations of NEFA and BHB are associated with several transition-related diseases, such as ketosis, displaced abomasum and metritis (LeBlanc et al., 2005; Duffield et al., 2009; Paiano et al., 2021). β-hydroxybutyric acid is known to be a suitable indicator for (sub)clinical ketosis (van der Drift et al., 2012), and decrease in body condition score (BCS) in early lactation is often used as indicator for the degree of NEB (Roche et al., 2015).

Cow health and welfare during the transition period may be enhanced if simple, non-invasive disease detection tools for postpartum diseases (e.g., ketosis) or NEB were available (Elliott-Martin et al., 1997). A few decades ago, it was already shown that non-invasive breath analysis was a suitable technique to detect ketosis at an early stage by sampling the exhaled breath of the cow and analyzing it using spectrometer analysis (Dobbelaar et al., 1996; Elliott-Martin et al., 1997; Mottram, 1997). More advanced electronic devices have been recently developed for detecting volatile organic compounds from breath samples obtained by positioning a funnel in front of the cow’s muzzle (Ali et al., 2022). Next to disease detection, measurement of gas emissions in cows is of interest, as mainly CH_4_ production is known to play an important role in global warming (Della Rosa et al., 2021). A frequently used, non-invasive way to asses the methane production of individual cows is the sniffer method, where the air near the animal’s nostrils is sampled through a fixed tube in a concentrate feeder or automatic milking system and gas concentrations are measured (Difford et al., 2018; Oikawa et al., 2022).

Insight into the metabolism of animals can also be captured using non-invasive measurements of exhaled breath, by the calculation of respiratory ratios like the Respiratory Exchange Ratio (RER). The RER is the ratio between the metabolic production of carbon dioxide (CO_2_) and the uptake of oxygen (O_2_) (Ramos-Jiménez et al., 2008). The RER is commonly used to indirectly determine the relative contribution of carbohydrates and lipids to overall energy expenditure (Simonson and DeFronzo, 1990; Ramos-Jiménez et al., 2008). A high RER (around 1) indicates that carbohydrates are the predominant source of energy, whereas a low RER (around 0.7) suggests that lipids are the main energy source used (Simonson and DeFronzo, 1990; Pendergast et al., 2000; Ramos-Jiménez et al., 2008). (Reynolds et al., 2019) found a RER around 1 in Jersey cows that were around 80 DIM, and (Yan et al., 1997) found a decrease in RER from 1 to 0.7 after 72 h of fasting in dairy cows. Hence, during a postpartum NEB when feed intake is low and body fat mobilization is high, a low RER is to be expected in dairy cows.

Methane carbon dioxide ratio (MCR; CH_4_/CO_2_) was suggested as a respiratory ratio to express the efficiency of microbial fermentation of the feed by ruminants (Madsen et al., 2010). MCR directly describes the proportion of the C excreted that is not metabolized to CO_2._ Daily production of CO_2_ and CH_4_ in the rumen is variable and is affected by dietary components, total amount of dry matter intake, level of milk production, stage of lactation, breed, rumen functioning (Negussie et al., 2017), and time between feeding and measurement (Crompton et al., 2011; van Lingen et al., 2017). MCR is therefore often used to find feed sources or specific cows with the most efficient conversion of energy and the lowest production of CH_4_ (Madsen et al., 2010).

Improvement of non-invasive detection of cows at risk for diseases can lead to a decrease in health and welfare problems, especially when the risk for postpartum diseases can already be predicted based on observations, such as exhaled breath composition, detected in the prepartum period. At this moment, it is not known if the respiratory ratios differ as a result of the health state of dairy cows, and whether measurements of exhaled breath could be used as indicator to detect dairy cows at risk for diseases. Hence, the objective of this case study was to investigate if respiratory ratios derived from gas concentrations (O_2,_ CO_2,_ and CH_4_) measured in exhaled breath of individual cows during their visits to a concentrate feeder measured from 2 wk prepartum until 6 wk postpartum could be used as indicators to detect dairy cows at risk for impaired postpartum health. The total deficit score (TDS) as previously developed by van Dixhoorn et al. (2018, 2023) was used as integral parameter for postpartum health. The TDS is calculated based on the combination of severity and duration of different clinical responses and was suggested to better reflect the total postpartum disease load compared with evaluating the presence or absence of one specific disease (van Dixhoorn et al., 2023). The TDS is especially developed for research practices, but may give valuable insights into the relation between integrated postpartum health and exhaled breath composition in dairy cows.

## Materials and Methods

The established principles of laboratory animal use and Dutch laws related to animal experiments were adhered to in this study. The Wageningen University Animal Care and Use Committee (Lelystad Department) approved the experiment under protocol number AVD401002016749.

### Animals, housing, and diet

The present study was conducted between July 2017 and April 2018 at two commercial dairy farms located in the Netherlands. A total of 57 Holstein-Friesian dairy cows were monitored from 2 wk prior to the expected calving date until 6 wk after actual parturition. Cows enrolled in the study based on the expected day of parturition. Cows were used in the experiment when they showed no clinical signs of illness prior to parturition and when a complete dataset until 6 wk after calving was available. Two cows were excluded from analysis due to missing data, which resulted in a dataset of 55 cows (20 cows of farm 1, 35 cows of farm 2). At both farms, dry cows and lactating cows were housed separately. All cows were kept in cubicles, and a straw bedded maternity pen was present. Cows were moved to the maternity pen with the first signs of parturition. One to 3 d after calving, the cows were (re)introduced in the lactating herd and stayed there during the entire lactation. Water was provided ad libitum on both farms.

Farm 1 had 75 cows with an average production of 8900 kg milk per year (with 4.53% fat and 3.52% protein). Lactating cows were milked twice daily at 5:45 AM and 5:00 PM, and the basal ration was fed once per day at 1:30 PM. The basal ration for lactating cows consisted of grass silage, oilseed rape straw, maize silage, and minerals (net energy for lactation: 7.0 MJ/kg of DM). Dry cows were fed the basal ration once every 2 d. The basal ration for dry cows consisted of grass silage, wheat straw, maize, and minerals (net energy for lactation: 5.8 MJ/kg of DM). Concentrate was provided every day and separately from the basal ration in individual concentrate feeders for both lactating and dry cows. Two weeks prepartum all cows received 2 kg of concentrate per day. Postpartum concentrate quantity depended on lactation stage, and increased stepwise according to the Dutch standard feedstuff table (min 3,5 kg/d and max 8 kg/d; CVB, 2016). The basal ration was pushed to the feeding fence at 5:40 AM, 4:55 PM, 8:00 PM, and 10:30 PM. Feed residues were removed from the feed bunk before each new feed delivery. Lights were turned on at 5:45 AM, and nightlights were turned on at 10:30 PM.

Farm 2 had 100 cows with an average production of 9430 kg milk per year (with 4.35% fat and 3.55% protein). Lactating cows were milked twice daily at 6:00 AM and 5:45 PM, and the basal ration was fed once per day at 8:30 AM. The basal ration for lactating cows consisted of grass silage, maize silage, and brewer’s grain (net energy for lactation: 6.3 MJ/kg of DM). Dry cows were fed the basal ration once every 2 d. The basal ration for dry cows consisted of wheat straw, maize, and brewer’s grain. Concentrate was provided every day and separately from the basal ration in individual concentrate feeders for both lactating and dry cows. Two weeks prepartum, all cows received 1 to 2 kg of concentrate per day. Postpartum concentrate quantity depended on lactation stage, and increased stepwise according to the Dutch standard feedstuff table (max 8 kg/d; CVB, 2016). The feed was pushed to the feeding fence at 5:30 PM. Feed residues were removed from the feed bunk before each new feed delivery. Lights were turned on at 5:15 AM and turned off at 10:15 PM.

### Clinical assessment

Clinical assessment of all cows was performed as previously described (van Dixhoorn et al., 2023). In short, cows were scored clinically by trained veterinarians twice weekly from 2 wk prepartum until 6 wk after parturition. The veterinarians scored 19 clinical signs (including BCS) and used measurements and cut-off values as described (Hajer et al., 2011). When the veterinarian diagnosed a disease (retained placenta, metritis, mastitis, lameness, displaced abomasum, respiratory infection, milk fever, diarrhoea), the specific disease was reported on top of the clinical scoring. Disease treatments were performed according to the standard farm routines. Interobserver variation between the trained veterinarians was verified every 4 mo.

### Blood sample collection and analysis

Blood samples were collected twice weekly from the coccygeal vein and directly tested for BHB with a handheld device (FreeStyle Ketone Test, Abbott Laboratories, The Netherlands). Additional blood samples were collected in the first and fifth week after calving in 10 ml sterile serum tubes (Vacutainer, Becton Dickinson, Franklin Lakes NJ). All samples were taken in the morning after milking, but before feeding.

Samples were submitted for analysis to a veterinary laboratory (Royal GD, Deventer, the Netherlands). From these blood samples, clinical-chemical parameters were assessed using a routine chemistry analyzer (UniCel DxC 600 Synchron Clinical System (Beckman Coulter)). Results were evaluated according to the quality control procedures of the laboratory (meeting NEN-EN-ISO 9001:2015 requirements). Test procedures for all parameters (except for calcium, magnesium, and haptoglobin) were NEN-EN-ISO/IEC 17025:2017 accredited by the Dutch Accreditation Council (RvA). Colorimetric methods were used to analyze serum concentrations of calcium (Calcium testkit from Randox Laboratories Ltd), phosphorus (ammonium-molybdate method; PHS reagent from Beckman Coulter), magnesium (MG reagent from Beckman Coulter), total bilirubin (dimethylsulphoxide method; Bilirubin (Total) test kit from Randox Laboratories Ltd), haptoglobin (“PHASE” Haptoglobin Assay; Tridelta Development Limited), total protein (Biuret method; Total Protein test kit from Human Diagnostics), and albumin (Bromocresol Green method; Albumin test kit from Randox Laboratories Ltd). The albumin:globulin ratio of the samples was subsequently calculated from the total protein and albumin concentrations (total globulins = total protein minus albumin). Enzymatic methods were used to analyze serum concentrations of urea (urease method; Urea testkit from Human Diagnostics), NEFA (Wako NEFA-HR(2) test kit; FUJFILM Wako Chemicals Europe GmbH), and BHB (D-3-Hydroxybutyrate (Ranbut) reagent from Randox Laboratories Ltd.). Aspartate aminotransferase (AST; GOT (ASAT) test kit from Human Diagnostics) and gamma-glutamyltransferase (GGT; gamma-GT test kit from Human Diagnostics) concentrations in serum were analyzed using enzymatic methods according to the International Federation of Clinical Chemistry (IFCC) reference procedures for the measurement of catalytic activity concentrations of enzymes at 37 °C. Interleukin-6 concentrations in serum were analyzed using an AlphaLISA Bovine IL-6 Detection Kit (PerkinElmer) following the kit’s instructions. Inter-assay coefficients of variation were < 10% for all assays.

### Total Deficit Score (TDS) calculation

TDS were calculated as previously described (van Dixhoorn et al., 2023), and were used as measures for postpartum health of individual cows. Briefly, results from clinical findings, disease diagnosis, and postpartum serum variables (week 1 and week 5) from the first 6 wk after calving were used for TDS calculations. Four different TDS scores were calculated based on clinical values and serum values related to metabolic stress, inflammation, and locomotion: TDS Total, TDS Metabolic, TDS Inflammation, TDS Locomotion (for more details see Supplementary Table S1a, b). In addition, the postpartum serum values contributed to TDS when they were below or above specific cut-off values. Based on specific serum values, TDS Metabolic was sub-divided into TDS scores related to liver function (TDS Liver) and macro-mineral shortage (TDS Macro-minerals). Clinical findings that deviated from normal were counted as one point of the TDS (dimensionless) per sampling moment during the first 6 wk after calving. When the veterinarian diagnosed the cow as ill, the specific diagnosis was reported, and two points were assigned to the respective TDS category. In addition, treatments related to the disease diagnosis received two points. Each corresponding deviating serum value at the two sampling moments (week 1 and 5 after parturition) received 6 TDS points. Finally, all scores were then summated per TDS category for each cow, resulting in one final TDS for each individual cow per TDS category. A high TDS in a specific category indicates that many deviating findings were observed related to this category in the first 6 wk after parturition in a particular cow, indicating a higher risk for impaired postpartum health compared with cows with a low TDS in this category.

### Categorization of BCS decrease, BHB, and TDS scores

Based on the reduction in BCS during the first 6 wk after calving, cows were assigned to one of two groups being BCS decrease Low (with a decrease in BCS of 1 point or less) or BCS decrease High (a decrease in BCS of 1.5 points or more). Based on the BHB serum values, cows were assigned to one of two BHB groups being BHB Low (all BHB measurements < 1.2 mmol/L or with a maximum of 2 times a BHB value of 1.2 mmol/L) or BHB High (BHB > 1.2 mmol/L in at least one sample or more than two times a BHB level ≥ 1.2 mmol/L).

**Figure 1.**
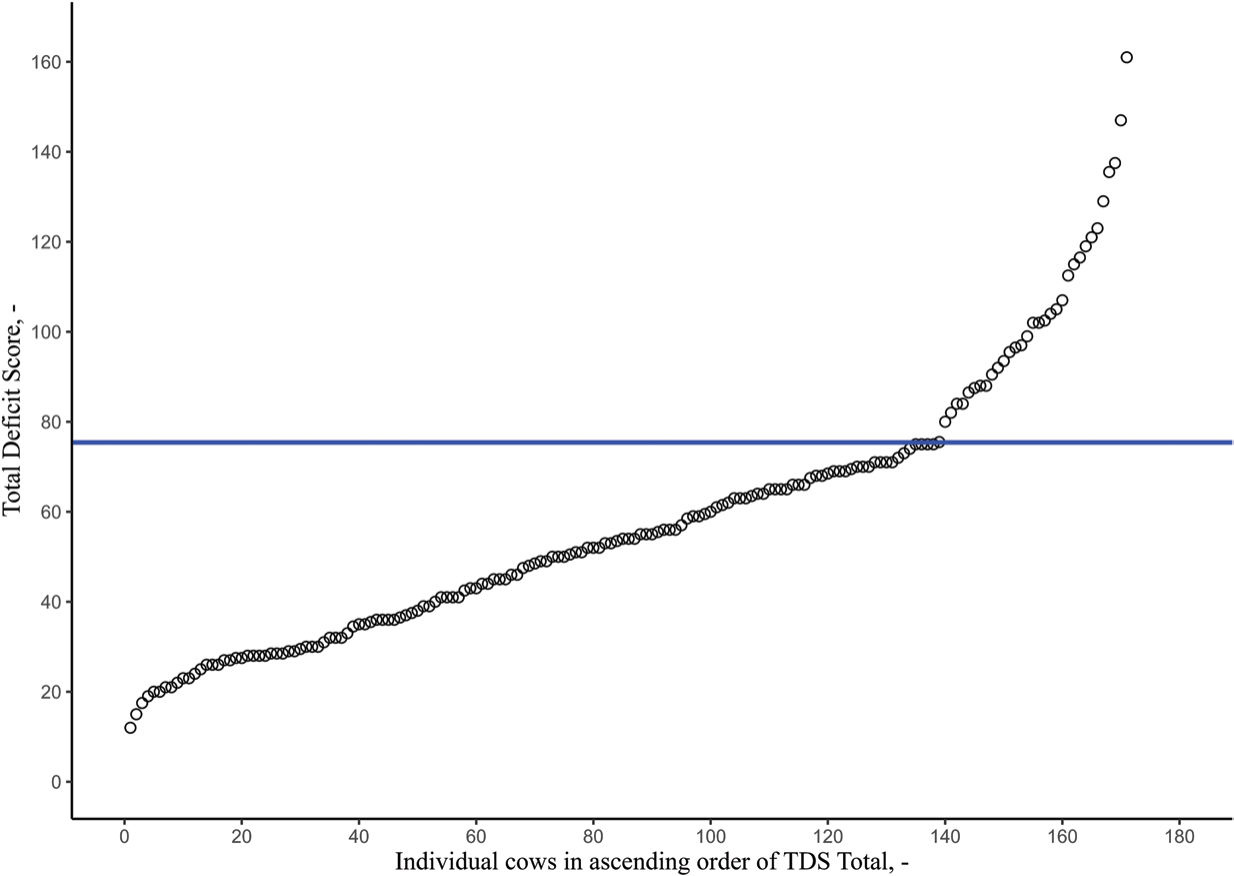
The piecewise linear analysis of the plotted Total Deficit Score (TDS) values of 171 cows (black circles; culled cows excluded). The breakpoint was found at TDS of 75.9 (blue horizontal line).

Cows were assigned to either a TDS Low or TDS High group per TDS category based on cut-off values per TDS category (Table 1). Per TDS category, the cut-offs were based on piecewise linear analysis of the TDS value in a specific category of all cows when plotted in ascending order. The method to assess the cut-off point for TDS Total is shown in Figure 1 as an example. This piecewise linear analysis looks for linear parts in a graph and calculates the cutoff point when the slope changes (indicated by the blue line in the plot). The ascending plotted TDS showed a gradual increase until the TDS increases more drastically. This drastic increase was previously indicated as the tipping point at which cows enter into a destructive feedback loop as the result of intertwined components of metabolic stress of altered nutrient metabolism, dysfunctional inflammatory responses, and oxidative stress during the transition period (Sordillo and Mavangira, 2014; van Nes et al., 2016; Van Dixhoorn et al., 2018; van Dixhoorn et al., 2023). With this method, for every TDS category, a TDS cutoff point was determined and was used to assign cows to a TDS Low or TDS High group per TDS category. The number of cows in the category Low or High per indicator, and contribution to category High per farm are shown in Table 2.

**Table 1.** Cut-off values per Total Deficit Score (TDS) category to assign individual cows to the High or Low TDS category. Cut-off values were based on piecewise linear analysis and descriptive statistics per TDS category of 55 dairy cows in 2 commercial farms.

		Descriptive statistic per TDS category			
	Cut-off value	Mean	Median	Min	Max
TDS Total	75.9	58	52	12	147
TDS Inflammation	30.5	21	18	5	61
TDS Locomotion	7.2	13	8	0	59
TDS Metabolic	28.3	24	21	4	82
TDS Liver	19.7	14	12	0	64
TDS Macro minerals	23.8	17	16	3	70

**Table 2.** Number of cows per indicator category (IC; Low or High), and cows in IC High per farm of 55 dairy cows in 2 commercial farms.

	Number of cows per IC		Number of cows in IC High per farm	
Indicator	Low	High	Farm 1	Farm 2
BCS^1^	46	9	3	6
BHB^2^	46	9	3	6
TDS^3^ Total	46	9	2	7
TDS^3^ Inflammation	44	11	3	8
TDS^3^ Locomotion	25	30	7	23
TDS^3^ Metabolic	40	15	4	11
TDS^3^ Liver	43	12	4	8
TDS^3^ Macro-minerals	44	11	4	7

^1^BCS decrease Low (decrease in BCS ≤ 1) and BCS decrease High (decrease in BCS ≥ 1.5).

^2^BHB Low (all BHB measurements < 1.2 mmol/L or with a maximum of 2 times a BHB serum value of 1.2 mmol/L) and BHB High (BHB > 1.2 mmol/L in at least one sample or more than two times a BHB serum level ≥ 1.2 mmol/L).

^3^Total Deficit Score (TDS) cut-off values per category were based on piecewise linear analysis (Table 1).

### Breath analysis

**Figure 2.**
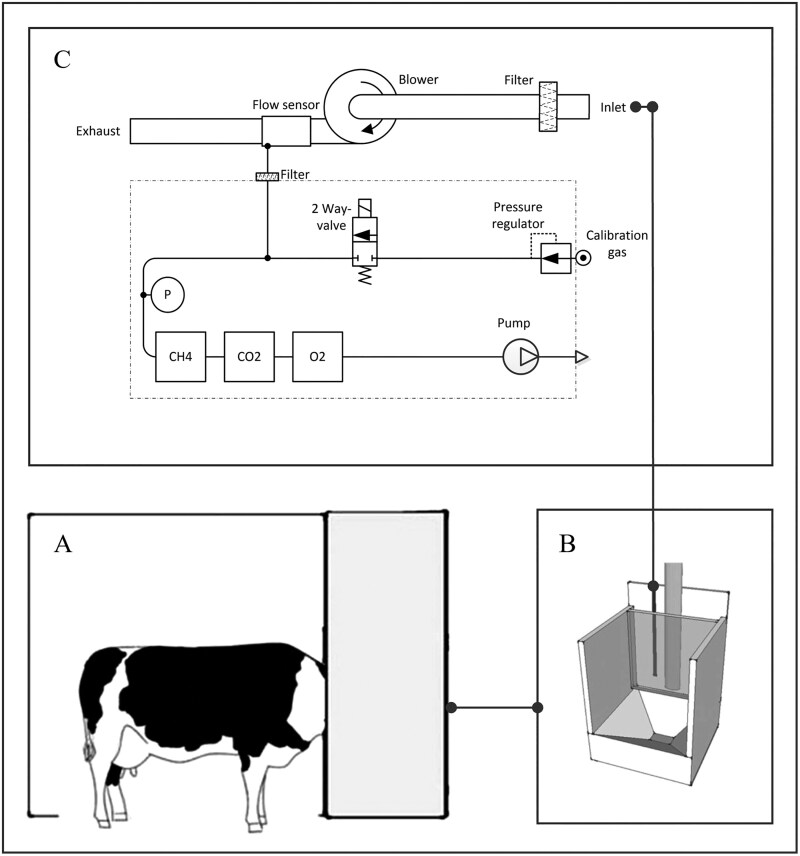
Schematic overview of a cow in the concentrate feeder (A). The cows’ breath flows in the air tube at the back of the feeding bin (B). The air tube is connected to the air inlet of the breath analysis device (C) where a flow sensor measures O_2_, CO_2_ and CH_4_ concentrations from a small air sample, after which the breath is exhausted from the device again. P represents the pressure sensor (scheme adapted from Garnsworthy et al. (2012b)).

A breath analysis device (Veterinary Metabolism System, Relitech, the Netherlands) was used to measure the concentrations of O_2_, CO_2_ and CH_4_ (vol%) in excess air sampled from the concentrate feeder with a flow of 200 L/min (± 50 L/min). The devices were installed in concentrate feeders present on the farms, resulting in respiration measurements during each visit of the cows to the feeder. A schematic overview of the breath analysis device installed in the concentrate feeder is shown in Figure 2 (adapted from (Garnsworthy et al., 2012b)). In short, a sample of the cows’ breath flows in the air tube at the back of the feeding bin. The air tube is connected to the air inlet of the breath analysis device, where a blower with controllable speed pulls air from the concentrate feeder through a dust filter, after which the air is exhausted through a flow sensor into the air again. Right behind the flow sensor a small sample of air is drawn through the analyzer system by a pump, where the gas concentrations of, and O_2_, CO_2_ and CH_4_ are measured with a sample rate of 1 Hz. A dust and bacterial filter is placed in front of the analyzer to avoid contamination of the air sample circuit. A pressure sensor right before the gas sensors (“P” in Figure 2) controls the air in the analyzer circuit, and can detect a congested filter and/or pump malfunction based on pressure changes. Here, air flow, air temperature, and pressure were measured with a sample rate of 1 Hz. All gas devices were calibrated every 3 wk or earlier. To enable calibration of the gas sensors, a two-way valve together with a calibration gas bottle is included in the air sample line. The two-way valve may be activated allowing surplus calibration gas to flow through the analyzer system. The O_2_ sensors were renewed half way through the experiment, due to degradation of the sensors. Degradation of the CH_4_ and CO_2_ sensors was negligible, and replacement was not necessary. Background gas concentrations were assessed by the sensors during periods that no cow was present in the concentrate feeder.

Five devices were available and these were first installed at farm 1 (measurements from July 2017 till the beginning of December 2017) and subsequently at farm 2 (measurement from December 2017 till the end of April 2018). One device was placed in the concentrate feeder of the dry cows, and the other four in the concentrate feeders for the lactating cows. Cows could enter the concentrate feeder all day and night. At each entry to the concentrate feeder, breath measurements took place. A radio frequency identification (RFID) reader was placed in the concentrate feeder that recognized individual cows by an RFID in their transponder when they entered the concentrate feeder. This was used to decide if a cow could receive concentrates. The start and end time per visit (hh:mm:ss) was recorded by the RFID reader and was used to link breath concentration measurements to individual cows. When the maximum amount of concentrates was consumed on a day, cows were able to enter the concentrate feeder without concentrate provision, and breath measurements still took place.

To ensure only quality measurements were included, visits were selected for respiration characteristic calculations if they contained at least 20 s of gas concentration data meeting quality criteria, regardless of total visit duration. The total duration of a visit to the concentrate feeder was not used as criterion because during a visit, a cow did not necessarily put her head in the feeding bin, and hence not during every visit reliable measurements took place. Preliminary analysis showed that a time window of at least 20 s resulted in reliable measurements. Raw data were filtered by using the following quality criteria: A time window of data was defined as valid if all samples in this window met the following gas concentration criteria: [CO_2_] ≥ 0.5% and [O_2_] ≤ 20.4%. For calculating the RER during a valid visit, an extra filter was applied: [CH_4_]/[CO_2_] ≤ 0.05 to discard samples that were influenced by eructation caused by rumen contractions. For each visit, the longest time window was taken that met these criteria and only the data of this window were used for analysis. Visits from which the longest valid time window was less than 20 s were discarded. Mean gas concentration per selected time window was calculated, and subsequently, a mean daily gas concentration per cow was calculated. Data from 2 cows were not used for analysis, due to missing values and low-quality measurements. This resulted in a final data set of 2030 mean daily records that were used for analysis, including 55 cows, 11 cows from parity 1, 27 cows from parity 2 and 3, and 17 cows from parity 4 or higher. The average number of visits per cow per day to the concentrate feeder (≥ 20 s) was 3.02 ± 1.90 with a minimum of 1 and a maximum of 13 visits per cow per day. The average duration per visit was 6.5 min (range 2 to 45 min).

For further analysis, the following three respiratory ratios were calculated per visit, corrected for background air concentrations, and averaged per day per cow:

- **Respiratory exchange ratio (RER)** defined as the ratio between CO_2_ and O_2_ concentrations (CO_2_/O_2_), excluding measurements influenced by ructus interruption
- **CH**_**4**_ **exchange ratio (MER)** defined as the ratio between CH_4_ and O_2_ concentrations (CH_4_/O_2_), including measurements when ructus had likely occurred.
- **CH**_**4**_ **-to-CO**_**2**_ **ratio (MCR)** defined as the ratio between CH_4_ and CO_2_ concentrations (CH_4_/CO_2_) including measurements when ructus had likely occurred.

RER indirectly determines the relative contribution of carbohydrates and lipids to overall energy expenditure (Ramos-Jiménez et al., 2008). MER indirectly determines the relative contribution of enteric fermentation to overall energy expenditure. MCR expresses the efficiency of microbial fermentation of the feed by ruminants (Madsen et al. 2010).

### Statistical analysis

All statistical analyses were performed using SAS University Edition (2.8.1 9.4 M6; SAS Institute Inc.). Daily mean gas concentrations and respiratory ratios were calculated per cow based on the data per visit. Descriptive statistics were calculated for each breath component (O_2_, CO_2_, CH_4,_ RER, MER, and MCR) based on the daily averages per breath component. For further statistical analyses, only respiratory ratios (RER, MER, MCR) were used. To evaluate normality of residuals, a normality test was performed (PROC UNIVARIATE) where skewness between −1 and 1 and kurtosis between −2 and 2 were used as criteria for normality. For analysis, RER was transformed by a natural logarithm, and MER was transformed by a square root to obtain normally distributed residuals. Statistical models are described below. Tukey’s studentized range tests were performed to investigate between-group differences when more than two groups were present.

To evaluate overall effects of parity and lactation stage on respiratory ratios, a mixed model was used (PROC MIXED). Any breath component ratio was included as dependent variable. Cow nested in farm was used as repeated subject. Parity group (parity 1, parity 2, or 3, parity ≥ 4), lactation stage (2 wk before calving [prepartum], 1 to 21 d in milk (DIM), 22-42 DIM), and their 2-way interactions were included as fixed effects.

To evaluate the relationship of TDS Total values with the decrease in BCS, a mixed model was used (PROC MIXED). The TDS Total value was used as dependent variable. BCS decrease group (Low or High), parity group (parity 1, parity 2 or 3, parity ≥ 4), and their 2-way interactions were used as fixed effect and farm as random effect.

Next, differences in breath composition were tested between the indicators (e.g., decrease in BCS, BHB serum values, and different TDS scores) during the three lactation stages (2 wk before calving [prepartum], 1-21 DIM, 22-42 DIM). A mixed model (PROC MIXED) was used, where the dependent variable was one of the breath components. Cow nested in farm was used as repeated subject, day relative to calving as repeated measurement, and parity group (parity 1, parity 2, or 3, parity ≥ 4) as random effect. Lactation stage, one of the indicators, and their 2-way interaction were used as fixed effects.

## Results

### Descriptive statistics of breath components

**Figure 3.**
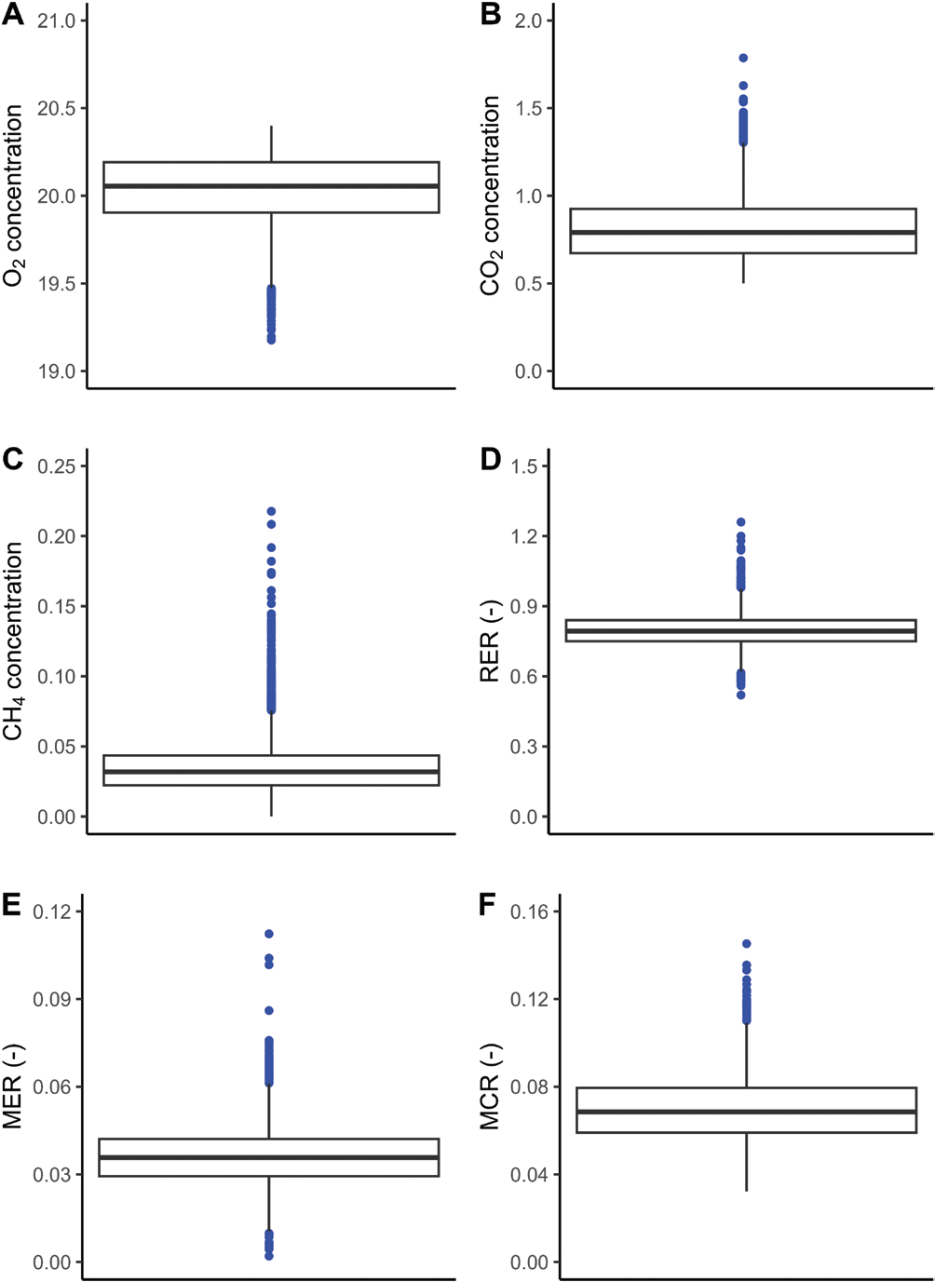
Mean (black horizontal line), first quartiles (black box), 90% interval (black line) and outlier values (blue dots) for daily average gas concentration of (**A**) oxygen - O_2,_ (**B**) carbon dioxide - CO_2_ and (**C**) methane - CH_4_ and the daily average (**D)** respiratory exchange ratio (RER), (**E**) CH_4_ exchange ratio (MER) and (**F**) CH_4_-to-CO_2_ ratio (MCR) measured in 55 cows on two commercial dairy farms in the Netherlands.

The distribution of the gas concentrations of O_2_, CO_2,_ and CH_4_ as well as the three respiratory ratios over the study period, is shown in Figure 3. Mean ± SD concentrations of O_2_, CO_2_ and CH_4_ were 20.0 ± 0.20, 0.81 ± 0.18 and 0.03 ± 0.02 vol% respectively. Mean ± SD of RER, MER, and MCR were 0.80 ± 0.01, 0.036 ± 0.01, and 0.07 ± 0.01, respectively. Respiratory ratios RER, MER, and MCR were used for further analysis.

**Figure 4.**
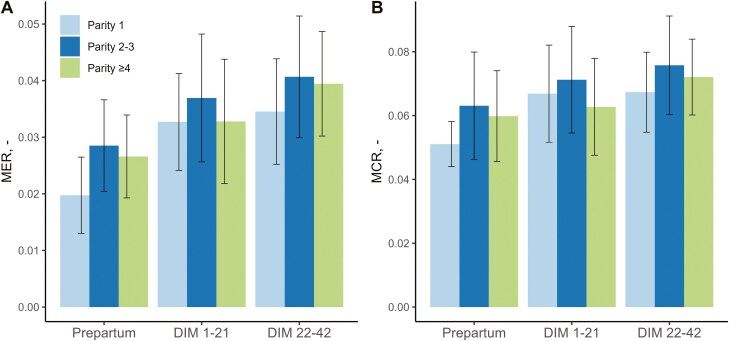
(**A**) Methane exchange ratio (MER) and (**B**) CH_4_-to-CO_2_-ratio (MCR) per lactation stage (prepartum = 2 wk before calving; DIM 1-21; DIM 22-42) and parity group of 55 cows on two commercial dairy farms in the Netherlands (mean ± SD).

Mean of respiratory ratios of the cows were tested for the effect of lactation stage and parity, and their 2-way interaction (Table 3). For RER, only an effect of lactation stage was found, where RER was higher in the prepartum period compared with DIM 1-21 and DIM 22-42. For MER and MCR also an interaction effect of lactation stage × parity group was found (Figure 4). For all three lactation stages, MER and MCR were highest for cows of parity 1. MER and MCR gradually increased from the prepartum period to DIM 22-42.

**Table 3.** Least square means ± SEM of the respiratory exchange ratio (RER), CH_4_ exchange ratio (MER) and CH_4_-to-CO_2_ ratio (MCR) per lactation stage and *P*-values for the effects of lactation stage (LS)^1^, parity (P)^2^ and their interaction (LS*P) based on 55 cows on two commercial dairy farms in the Netherlands^3^

	Lactation stage				*P-*value		
Variable	Prepartum	DIM 1-21	DIM 22-42	SEM	LS	P	LS*P^4^
RER	0.82^a^	0.79^b^	0.79^b^	0.008	<0.001	0.14	0.54
MER	0.025^c^	0.034^b^	0.038^a^	0.001	<0.001	<0.001	0.001
MCR	0.059^b^	0.067^a^	0.071^a^	0.003	<0.001	<0.001	0.02

^a-b^Values with different superscripts in the same row differ (*P *< 0.05).

^1^Lactation stages where: prepartum (2 wk before calving); DIM 1-21; DIM 22-42.

^2^Parity groups where: parity 1; parity 2-3; parity ≥ 4.

^3^RER was log-transformed and MER was square rooted for analysis, but non-transformed values are shown in Table 3.

^4^Significant (*P* < 0.05) interactions between LS × P for MER and MCR are shown in Fig 4.

### Associations between TDS Total and decrease in BCS

We evaluated associations between TDS Total values and decrease in BCS, parity group, and their 2-way interaction. An association was found between the BCS decrease group and TDS Total values (*P < *0.05) independent of parity, where cows with a large decrease in BCS had higher TDS Total values than cows with a low decrease in BCS. In addition, the TDS total value tended to be higher for cows of parity **≥ **4 than for younger cows (*P *= 0.08).

### Associations between postpartum decrease of BCS and BHB serum values on breath composition

**Figure 5.**
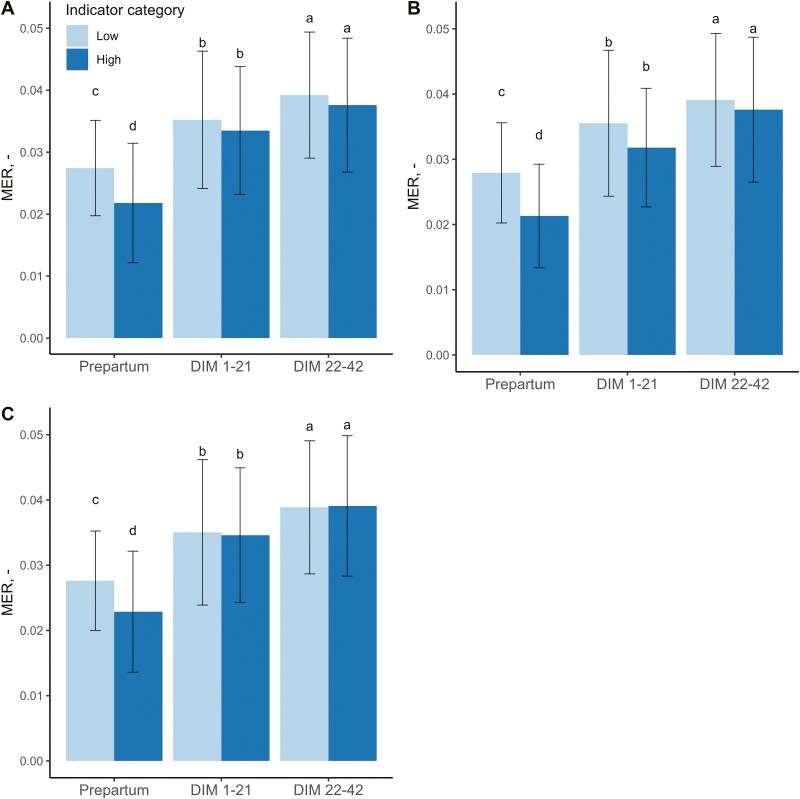
The methane exchange ratio (MER) for (**A**) BCS decrease (Low (decrease in BCS ≤ 1) and High (decrease in BCS ≥ 1.5)) and Total Deficit Scores (TDS) (**B)** Total and (**C**) Inflammation (Low and High) in different lactation stages (prepartum = 2 wk before calving; DIM 1-21; DIM 22-42) of 55 cows on two commercial dairy farms in the Netherlands (mean ± SD). ^a- d^ Mean values with different superscripts differ significantly (*P* < 0.05).

Associations between postpartum decrease of BCS, BHB serum values on RER, MER, and MCR are represented in Table 4. Cows with a large decrease in BCS during the first 6 wk after calving had lower RER compared to cows with only a limited decrease in BCS. For MER, an interaction between BCS and lactation stage was found, and post hoc comparison showed a significantly lower MER value in the prepartum period for the cows with a high decrease in BCS after calving compared with the cows with a low decrease in BCS after calving (Figure 5). No difference in MER was seen between the BCS decrease High and BCS decrease Low after calving, and no significant relationships were found between BCS group and MCR. Cows with low BHB serum values had a higher RER and a lower MCR as compared with cows with high BHB serum values, but no interaction effects between BHB group and lactation stage were present.

**Table 4.** Respiratory exchange ratio (RER), CH_4_ exchange ratio (MER), and CH_4_-to-CO_2_ ratio (MCR) per indicator category (IC; Low or High)^1^ for 6 TDS categories, BCS decrease, and BHB in 55 dairy cows on two commercial farms^3,4^. Significant effects of IC and the interaction with lactation stages (LS)^2^ are also given.

				*P*-value^7^					*P*-value					*P*-value	
Item	RER^5^		SEM	IC	LS*IC	MER^5^		SEM	IC	LS*IC^6^	MCR		SEM	IC	LS*IC
IC	Low	High				Low	High				Low	High			
BCS	0.81^a^	0.79^b^	0.009	0.03	0.83	0.033^a^	0.030^b^	0.002	<0.01	0.02				0.90	0.40
BHB	0.81^a^	0.78^b^	0.009	0.01	0.16				0.13	0.09	0.065^b^	0.069^a^	0.003	0.02	0.92
TDS Total				0.12	0.06	0.035^a^	0.031^b^	0.002	<0.01	<0.01	0.069^a^	0.064^b^	0.002	<0.01	0.49
TDS Inflammation				0.91	0.09				0.22	<0.01				0.30	0.95
TDS Locomotion				0.19	0.98	0.034^a^	0.032^b^	0.002	<0.01	0.31	0.069^a^	0.065^b^	0.002	<0.01	0.28
TDS Metabolic				0.36	0.10	0.033^a^	0.029^b^	0.002	<0.01	0.70	0.068^a^	0.062^b^	0.002	<0.01	0.28
TDS Liver	0.81^a^	0.79^b^	0.006	0.04	0.06	0.032^a^	0.029^b^	0.002	<0.01	0.12				0.13	0.51
TDS Macro-minerals				0.52	0.25	0.034^a^	0.032^b^	0.002	<0.01	0.88	0.067^a^	0.061^b^	0.002	<0.01	0.23

^a-b^Values with different superscripts in the same row differ (*P *< 0.05).

^1^Group cut-offs were based on: body condition score (BCS) decrease Low (decrease in BCS ≤ 1) and BCS decrease High (decrease in BCS ≥ 1.5); β-hydroxybutyric acid (BHB) Low (all BHB measurements < 1.2 mmol/L or with a maximum of 2 times a BHB value of 1.2 mmol/L) and BHB High (BHB > 1.2 mmol/L in at least one sample or more than two times a BHB level ≥ 1.2 mmol/L); Total Deficit Scores (TDS) Group cut-off values were based on piecewise linear analysis;.

^2^Lactation stages where: DP (2 weeks before calving); DIM 1-21; DIM 22-42.

^3^Least square means ± maximum SEM.

^4^Least square means that are missing are not shown because no significant model effect was found.

^5^RER was log-transformed and MER was square rooted for analysis, but non-transformed values are shown in Table 4.

^6^Significant (*P *< 0.05) interactions between LS × IC are shown in Figure 5.

^7^LS was always significant (*P* < 0.05), and therefore not shown in this table.

### Associations between TDS on breath composition

Associations between different TDS categories on RER, MER, and MCR are represented in Table 4. The RER was only associated with TDS Liver, with cows showing better liver function (fewer liver problems) having a higher RER compared with cows with poorer liver function (more liver problems), independent of lactation stage. Around calving, the MER was associated with TDS Locomotion, Metabolic, Liver, and Macro-Mineral, but no interaction effects with lactation stage were found. For each of these categories, the direction of the effect was the same as cows with a low TDS had a higher MER compared with cows with a high TDS. Cows that had less locomotion, metabolic, or liver problems had a higher MER compared with cows with more of these problems. Cows with a better macro-mineral balance had a higher MER as compared with cows with a worse macro-mineral balance.

Lactation stage was associated with the MER for TDS Total (*P *< 0.01) and TDS Inflammation (*P *< 0.01) (Table 4, Figure 5). The MER increased for TDS Total and TDS Inflammation as the lactation progressed. Cows with a better overall postpartum health and better inflammatory status had a lower MER during the prepartum period compared with cows with a worse overall postpartum health and inflammatory status in this period. No differences were found between groups during the postpartum period.

The MCR was associated with the TDS Total, Locomotion, Metabolic, and Macro-minerals, but no interaction effects with lactation stages were found. Cows with a low TDS Total (healthy cows) had a higher MCR as compared with the cows with a high TDS. Cows with less locomotion or metabolic problems and a better macro-mineral balance had a higher MCR as compared with cows that had more locomotion or metabolic problems and a worse macro-mineral balance.

## Discussion

In this case study, the relationships between respiratory ratios RER, MER, and MCR derived from non-invasive measurements of exhaled breath and different health aspects in dairy cows were evaluated from 2 wk prior to calving date until 6 wk after calving. In the current study, we related respiratory ratios to lactation stage, parity, degree of decrease in BCS, and different disease categories.

The non-invasive breath measurement technique used in the current study was comparable to the sniffer method (Huhtanen et al., 2015), and respiratory ratios were used to evaluate results, which was done previously (Oikawa et al., 2022; Uemoto et al., 2024). This method enables the evaluation of variation in exhaled breath composition caused by gas production and consumption by individual animals. The device used in our study could easily be applied to the concentrate feeders and could also be built in an automatic milking system, as previously suggested by Madsen et al. (2010). However, this sniffer-like method is considered less accurate compared to using a gas-flux quantification system (GreenFeed; C-Lock Inc. Rapid City, SD) (Huhtanen et al., 2015), or a respiration chamber where emissions from exhaled breath, manure, and urine of single cows are measured (Negussie et al., 2017). Sniffer methods often show high variation in gas concentrations between cows (Garnsworthy et al., 2012b; Bell et al., 2014a), likely due to the movement of the cow’s head, the distance of the muzzle to the air inlet, and the variability in mixing of air within the feeding bin (Huhtanen et al., 2015). In this study, the use of respiratory ratios, instead of gas concentrations per se, reduces the impact of the higher between-cow variation in measurements, under the assumption that the three involved gases mix and disperse in the same way after exhaling. However, the limitations of the current measurement method in combination with the relatively small sample size should be taken into consideration while further developing this method.

In the current study, a lower level of RER was found in the first 6 wk of lactation compared with the prepartum period. In early lactation, cows are in a stage of NEB, which is caused by the increase in the nutrient demand for milk production. If the energy need is exceeded by the energy supply via feed intake, the cows will start to mobilize body fat to meet high energy demands (Sundrum, 2015). This might explain the lower level of RER in the first 6 wk of lactation compared with the dry period, where relatively more carbohydrates are utilized (Kenny et al., 2012).

The MER and MCR differed between lactation stages and parities. Both MER and MCR gradually increased over the different lactation stages for all parity groups. This is a result of increasing feed intake and milk production at the start of lactation, resulting in higher CH_4_ production (Garnsworthy et al., 2012a; Haque et al., 2014). The MER was lower in primiparous cows during all lactation stages compared with multiparous cows. In addition, the MCR was lower for primiparous cows during the prepartum period and weeks 3 to 6 of lactation compared to multiparous cows. This is in line with other studies that found lower levels of CH_4_ production in primiparous cows compared with multiparous cows (Garnsworthy et al., 2012a; Fresco et al., 2023). Primiparous cows generally have a lower milk production and lower feed intake than multiparous cows (Azizi et al., 2009), thereby likely reducing CH_4_ production in these cows. In addition, CH_4_ emissions were also found to increase with body weight (Moraes et al., 2014; Antunes-Fernandes et al., 2016), and primiparous cows have generally the lowest body weight in the herd. The findings in the current study may suggest that parity-specific models are required when further developing non-invasive breath measurement as on-farm monitoring tool in the future, but evaluation on a broader set of farms is required to confirm these findings and to support external validity.

BHB serum values were related to breath composition in the current study, as cows with high BHB serum values had a lower RER than cows with low BHB serum values. Elevated BHB concentrations are often associated with increased risk for (sub)clinical ketosis (Duffield et al., 2009; Seifi et al., 2011). The low RER in cows with high BHB serum values indicated that these cows mobilized more fat as the predominant fuel source than cows with lower BHB serum values (Kenny et al., 2012). This was in line with previous research who found relationships between ketosis and exhaled breath composition (Dobbelaar et al., 1996; Elliott-Martin et al., 1997; Mottram, 1997). A recent study showed that a rise in blood BHB was related to a rise in breath acetone, but no differences were found between the breath acetone levels of ketotic and non-ketotic cows (van Erp-van der Kooij et al., 2023). They suggested to perform longitudinal breath measurements to capture within-cow changes in breath composition for disease detection. Breath composition also varies highly throughout the day, further supporting the need for longitudinal sampling of breath (Suntrup et al., 2020). This highlights the value of sampling breath in the concentrate feeder, which cows visit multiple times a day. However, individual variation in the number of visits to the concentrate feeder between cows and its impact on the breath measurements should be further investigated. The role of social dominance could be of specific interest in this respect since it may affect feeding behaviour in cows, especially when feeding spaces are limited (Huzzey et al., 2006; DeVries, 2019).

Our results show that the cows with the largest decrease in BCS had the highest TDS Total scores, and were thus most at risk for disease as a results of high degree of body fat mobilization due to NEB. This was expected as body fat mobilization is often associated with NEB and ketosis, which are risk factors for postpartum diseases like metritis, cystic ovarian, lameness, and displaced abomasum (Walsh et al., 2007; Roberts et al., 2012; Suthar et al., 2013). However, cows with a high TDS Total score did not always have high BHB values, and cows with a low TDS Total score did sometimes have high BHB values. This may, on the one hand, partly be attributed to the small proportion of the cows that were diagnosed with high BHB serum values in this study. On the other hand, this confirms previous findings that high BHB serum values are not always associated with postpartum disease (Chapinal et al., 2011) but may indicate adaptation to high milk yield in early lactation (Mann and McArt, 2023). In addition, it should be noted that many plasma metabolites, such as BHB and NEFA follow a diurnal rhythm influenced by time of feeding (Meier et al., 2010; Niu et al., 2014). In this study, the time of feeding, and consequently, the time of blood sampling relative to the feeding time, was not fully aligned between the two farms, which may have influenced the TDS calculations. However, although fresh feed was offered at a different time at each farm, feed was regularly pushed up to the feeding fence between daily feed deliveries, effectively resulting in ad libitum feed availability at both farms. A random farm effect was included in the statistical models, which is assumed to account for most of the variation caused by differences in farm management. Nevertheless, there remains a need for a more detailed evaluation of the effects of feeding time and feed intake, not only on components of our TDS, including blood parameters, but also on breath measurements.

The RER was indicative for TDS Liver, independent of lactation stage. Cows that had more liver problems based on a high TDS Liver had a lower RER compared with cows with less liver problems. The lower RER in cows with more liver problems indicates that these cows mobilized more body fat as an energy source compared with cows with less liver problems. Liver problems, especially fatty liver, develop primarily in the periparturient period because of insufficient nutrient uptake and hormonal changes (Bobe et al., 2004). Liver lipid accumulation occurs when body fat stores are mobilized and release NEFAs into blood, which, to a considerable part, reach the liver (Grummer, 1993). If NEFA uptake exceeds the capacity of the liver to completely oxidize NEFAs to CO_2_, partial oxidation to form ketones or esterification to form triglycerides may result. Excessive production of ketone bodies can adversely affect animal behavior and performance (Grummer, 1993; Bobe et al., 2004). Cows with high BCS (≥ 4) in the dry period are especially at risk for liver problems as they typically have a greater decrease in feed intake after calving, causing a more severe NEB and excessive body fat mobilization (Stockdale, 2001). This is often associated with elevated serum levels of BHB, NEFA, aspartate aminotransferase, gamma-glutamyl transferase, and bilirubin (Bobe et al., 2004). These are the serum values that were included in our TDS Liver. Our results suggest that RER is mainly associated with excessive fat mobilization, and the TDS Liver might be a suitable read out to evaluate RER.

Feed intake is important in cow metabolism, as it affects rumen functioning and energy balance. When a NEB coincides with a reduced feed intake, the rumen functioning will be reduced, and RER may be affected (Kuhlmann et al., 1985; Bobe et al., 2004). In a recent study CO_2_ production and O_2_ consumption could contribute to feed intake prediction (Huhtanen et al., 2021), which is closely related to our RER. In the current study, cows with liver problems generally had a low RER, which was hypothesized to be related to a severe reduction in feed intake combined with a NEB. In future studies, individual feed intake of cows should be taken into account when interpreting RER in relation to cows’ health.

The MCR was previously suggested as an indicator for the efficiency of microbial fermentation of the feed by ruminants (Madsen et al., 2010), and MER indirectly determines the relative contribution of enteric fermentation to overall energy expenditure. In our study, lower levels of MER were related to several health problems. For cows suffering from liver problems, MER was lower compared with healthy cows. In addition, prepartum MER was lower in cows with a high TDS Total or TDS Inflammation than in cows with a low TDS Total or TDS Inflammation (as indicated by the interaction effect with lactation stage). Health disorders like metritis and retained placenta are often associated with decreased feed intake already in the prepartum period (Huzzey et al., 2007; Luchterhand et al., 2016). In general, CH_4_ production decreases with decreasing dry matter intake (Ellis et al., 2007; Ramin and Huhtanen, 2013), as rumen function and fermentation are reduced. In addition, cows with health problems may have a higher energy expenditure for maintenance due to the high energy demand of the immune system (Kvidera et al., 2017), thereby increasing O_2_ consumption. Hence, both CH_4_ production and O_2_ consumption may change in cows with health problems, thereby reducing MER possibly already during the prepartum period. This highlights the potential predictive value of non-invasive breath analysis techniques for cow health management purposes, and the use of breath composition measurements to predict the risk of postpartum disease already during the prepartum period. This approach is especially useful during the dry period as it allows for preventive health management at a time when health monitoring through non-invasive milk sampling is not possible. Previously, we have shown that also prepartum behavioral patterns of cows, recorded with the use of activity sensors, were significantly associated with postpartum health as indicated by the TDS (van Dixhoorn et al., 2023). Thus, we hypothesize that an integration of breath composition with other parameters, such as cow behavior, may further enhance the overall predictive value of prepartum measurements for postpartum health outcomes in dairy cows. Correspondingly, we suggest that further research into multivariable approaches for the development of cow health monitoring systems is urgently warranted.

Associations between MCR and health-related variables were less clear. Cows with locomotion, metabolic, or overall health problems had a lower MCR compared with healthy cows of these groups. In contrast, cows that suffered from ketosis had a higher MCR compared with cows without ketosis. This was unexpected, as ketosis is often closely related to other metabolic problems (Bobe et al., 2004). Factors like individual dry matter intake, milk production, diet composition, adaptation to lactational diet, and body weight strongly affect both CO_2_ and CH_4_ production (Johnson and Johnson, 1995; Antunes-Fernandes et al., 2016; Negussie et al., 2017; Huhtanen et al., 2021), thereby affecting MCR. These individual cow factors were not taken into account in the current study, and the results of the MCR should therefore be interpreted with caution. A recent study showed that exhaled volatile organic compounds, like acetone, propionate, and butyrate, match with daily patterns of CH_4_ and CH_4_ could therefore be a proxy for rumen volatile fatty acids (Islam et al., 2023). In future studies, we recommend to include more individual cow factors and generating more knowledge regarding rumen function, gas production, and cow health.

Another aspect that should be taken into account when measuring individual CH_4_ production per cow is the time lag between roughage feeding time and time of measurement (de Mol et al., 2024). The CH_4_ production typically shows a pattern with a peak at 1 to 2 h after feeding time depending on the amount of feed offered (Crompton et al., 2011), and after the peak the production gradually decreases again. Also, CO_2_ production follows a diurnal pattern, which is affected by feed intake (Kinsman et al., 1995; Bell et al., 2014b). Therefore, the moment of visiting the concentrate feeder influences the outcome of CO_2_ and CH_4_ production, and several breath measurements throughout the day are required to fully estimate CH_4_ production (Brask et al., 2015; Bell et al., 2018). It is expected that visits to the concentrate feeder are randomly distributed over the day, thus averaging out the effect of moment of sampling, and consequently on respiratory ratios. However, further research is needed to investigate the influence of the moment of breath measurement in relation to roughage feeding time on CO_2_ and CH_4_ production. This allows for better understanding and interpretation of the respiratory ratios.

The RER, MER, and MCR derived from non-invasive measurement of exhaled breath seem promising indicators for cow’s health and metabolic state, and may support early disease detection already during the prepartum period when cow monitoring is scarce and early interventions are possible. Although the TDS scoring system used in this case study is a comprehensive and detailed manner of evaluating postpartum health of dairy cows, thereby enhancing the statistical power of the study, findings of this case study should be confirmed using data from a larger number of cows. Also, the diurnal variation in breath concentrations in response to feeding and changes in feed composition should be further investigated. To allow for on-farm application, an accurate detection model for cows at risk for disease should be developed based on the exhaled breath analysis method with sufficient sensitivity and specificity. Potentially, an integration of several technologies, including sensor-based activity measurements, could improve early disease detection in dairy cows even more, thereby improving their health, welfare, and enhancing sustainable production.

## Conclusion

Respiratory ratios derived from non-invasive measurements of exhaled breath seem to be promising indicators to detect cows at risk for diseases during the transition period. The RER was related to body fat mobilization, as a lower RER was associated with a large decrease in BCS and more liver problems in the peripartum period. For many disease categories, MER was higher in healthy cows compared with diseased cows, but associations between MCR and health-related variables were less clear. Both MER and MCR results should be interpreted with caution, as CH_4_ production is affected by several factors like milk yield, feed intake, diet composition, rumen functioning, and body weight. In future studies, these variables should be taken into account to better understand the complex relationships between exhaled breath ratios, rumen function, health state, and production level. In addition, the lag between feeding time and sampling time should be examined in detail to assess its influence on CO_2_ and CH_4_ measurements.

## Supplementary Material

skaf234_suppl_Supplementary_Tables_S1

## Abbreviations

BCS: body condition score
BHB: β-hydroxybutyric acid
CH_4_: methane
CO_2_: carbon dioxide
DIM: days in milk
NEB: negative energy balance
LSM: least-square means
MCR: methane carbon dioxide ratio
MER: methane exchange ratio
NEFA: non-esterified fatty acids; O_2_, oxygen
RER: respiratory exchange ratio
RFID: radio frequency identification
TDS: Total Deficit Score
