# Supplementary material for: On-farm analysis of exhaled breath compounds as indicators for a postpartum health score in dairy cattle: a case study
Source: J Anim Sci. 2025 Jul 18;103:skaf234. doi: 10.1093/jas/skaf234 (PMC12342983; doi:10.1093/jas/skaf234)
Supplement: skaf234_suppl_Supplementary_Tables_S1 [file skaf234_suppl_supplementary_tables_s1.docx]

**Supplementary materials**

**Table S1a.** Overview of clinical observations and points assigned to the Total Deficit Score (TDS) categories: Total, Inflammation, Locomotion and Metabolic. Each suboptimal clinical value according to references was scored with 0.5, 1 or 2 points.

| **Clinical observation** | **Points** | **TDS** |
| --- | --- | --- |
| Ears cold | 1 if yes | Inflammation, Metabolic, Total |
| Excreta from nose | 1 if yes | Inflammation, Total |
| Jugular pulse visible above mid neck region | 1 if yes | Inflammation, Total |
| Rectal Temperature | 1 if T > 39.2  2 if T > 40 | Inflammation, Total |
| Breathing abnormal | 1 if yes | Inflammation, Total |
| BCS ^a^ | 1 if difference between BCS in dry period >1 | Metabolic, Total |
| Rumen visible when standing behind cow | 1 if yes | Metabolic, Total |
| Rumen fill weak | 1 if yes | Metabolic, Total |
| Rumen score^a^ | 0 if score 3-5; 1 if score = 1-2; 2 if score = 0; | Metabolic, Total |
| Udder edema | 1 if yes | Total |
| Udder score per quarter |  |  |
| Firm LF/RF/LB/RB^b^ | 0.5 if yes per quarter | Inflammation, Total |
| Red LF/RF/LB/RB^b^ | 0.5 if yes per quarter | Inflammation, Total |
| Abnormal uterus fill / excreta | 1 if yes | Inflammation, Total |
| Manure score^a^ | 2 if score =1, 1 if score is 2 or 5 | Metabolic, Total |
| Abnormal digestion | 1 if yes, | Metabolic, Total |
| Locomotion Score^a^ | 0 if score 1 or 2; 1 if score = 3-5 | Locomotion, Total |
| Lame LB, LF, RB, RF^b^ | 1 if yes per leg | Locomotion, Total |
| Cow is diagnosed ill | 2 if yes | assigned to specific TDS depending on disease |
| Treatment | 2 if yes | assigned to specific TDS depending on disease |

^a^ References used for interpretation of observations and score systems for BCS, manure score, locomotion and rumen score according to Royal GD, Deventer the Netherlands (Hajer et al., 2011).

^b^ LF: Left Front, RF: Right front, LB: Left Back, RB: Right Back

**Table S1b.** Overview of cut-off serum values resulting in a Total Deficit Score (TDS) per TDS categories (Inflammation and Metabolic, with Metabolic subdivided into Macro-minerals and Liver). Cut-off values of serum parameters for samples taken in week 1 and 5 are shown. Per sampling point (week 1 and week 5), a parameter exceeding the values as indicated in the last two columns, counted as 6 point in the TDS.

| **Parameter** | **Unit** | **TDS** | **Week 1** | **Week 5** |
| --- | --- | --- | --- | --- |
| Total protein | g/L | Inf^1^, Total | > 85 | > 85 |
| Total protein | g/L | Met^2^, Total | < 55 | < 55 |
| Albumin | g/L | Met, Total | < 31 | < 31 |
| Urea | mmol/L | Met, Total | < 3.3 | < 3.3 |
| Urea | mmol/L | Met, Total | > 6.6 | > 6.6 |
| NEFA^4^ | mmol/L | Met, Total | > 0.8 | > 0.4 |
| BHB^5^ | mmol/L | Met, Total | > 1.2 | > 1.2 |
| Calcium | mmol/L | Macro^3^, Met, Total | < 2.00 (day 0 – 1 )  < 2.20 (day 2 – 7) | < 2.20 |
| Magnesium | mmol/L | Macro, Met, Total | < 0.78 | < 0.78 |
| Phosphorus | mmol/L | Macro, Met, Total | < 0.9 | < 1.1 |
| AST^6^ | IU/L | Liver, Met, Total | > 115 | > 115 |
| GGT^7^ | IU/L | Liver, Met, Total | > 34 | > 34 |
| Total Bilirubin | µmol/L | Liver, Met, Total | > 7 | > 7 |
| Haptoglobin | g/L | Inf, Total | > 0.6 | > 0.3 |
| Interleukin-6 | ng/mL | Inf, Total | > 10 | - |
| Globulins (TP^8^-albumin) | g/L | Inf, Total | > 49 and TP < 85 and albumin > 31 | > 49 and TP < 85 and albumin > 31 |

^1^Inf : TDS Inflammation; Met^2^: TDS Metabolic; Macro^3^: TDS Macro-minerals; NEFA^4^: non-esterified fatty acids , BHBA^5^: β-hydroxybutyric acid, AST^6^: aspartate aminotransferase , GGT^7^: gamma-glutamyl transferase, TP^8^: Total Protein. Cut-off values of serum metabolites parameters were based on the upper and or lower limit of the reference intervals for the corresponding parameters as provided by veterinary laboratory of Royal GD (Deventer, the Netherlands), except for BHBA, NEFA, and calcium. The cut-off value for BHB was chosen based on the threshold for subclinical ketosis (Duffield et al., 2009), whereas the cut-off value for NEFA was chosen based on the threshold for an increased risk of early lactating culling, and clinical diseases (Ospina et al., 2010, Roberts et al., 2012, Ospina et al., 2013). The threshold for calcium was based on Kimura et al. (2006) and Martinez et al. (2012). The average DIM of blood sample collection were 1.8 ± 1.2 d and 29.8 ± 1.6 d for the first and fifth week sampling timepoints respectively.
